# DFVF: database of fungal virulence factors

**DOI:** 10.1093/database/bas032

**Published:** 2012-10-22

**Authors:** Tao Lu, Bo Yao, Chi Zhang

**Affiliations:** School of Biological Sciences, Center for Plant Science and Innovation, University of Nebraska, Lincoln, NE 68588, USA

## Abstract

Fungal pathogens cause various diseases for plant and animal hosts. Despite the extensive impact of fungi on human health and life, the threats posed by emerging fungal pathogens are poorly understood. Specifically, there exist few fungal virulence gene databases, which prevent effective bioinformatics studies on fungal pathogens. Therefore, we constructed a comprehensive online database of known fungal virulence factors, which collected 2058 pathogenic genes produced by 228 fungal strains from 85 genera. This database creates a pivotal platform capable of stimulating and facilitating further bench studies on fungal pathogens.

**Database URL:** http://sysbio.unl.edu/DFVF/

## Introduction

Fungal organisms comprise one of the most diverse kingdoms on Earth; the number of fungal species is estimated to be ∼1.5 million (1). Many fungal taxa are pathogens under certain conditions. For instance, ∼80 000 fungal taxa have pathogenicity on 56 000 vascular plant hosts, according to the Fungus–Host Distributions of US Department of Agriculture (http://nt.ars-grin.gov). Fungal pathogens have a broad spectrum of hosts, including plants and animals, and may cause death and disability in humans, yield loss in agricultural crops and even alteration of forest ecosystem dynamics. For example, fungi in the genus *Fusarium* cause a variety of blights, seedling disease, root rots or wilts on nearly all species of cultivated plants, and they strongly affect the agriculture economy. Virulence factors are the most important proteins in pathogens, such as toxin synthetic enzymes and secreted biodegradation enzymes (2), that permit them to evade the defense mechanisms of the host and, thus, cause diseases (3). Currently, the number of reported fungal virulence factors is limited, and many of the data are only available in the literature texts. Please refer to references (4–8) for reviews on fungal virulence factors. To maximize the value of this type of data, it is essential that a method of data storage and sharing should be implemented for efficient and effective data mining. To this end, we believe that the utilization of a web-based comprehensive database will significantly facilitate such activities. Currently, there exists only one database, PHI-base (9), for fungal pathogens. Although PHI-base collected pathogenic genes for all types of fungal and bacterial pathogens, it contained limited number of fungal virulence factors, and most of its records are about bacterial pathogens. In comparison, advanced databases and prediction tools similar to what we are proposing have been developed for many other bacterial pathogens and were proven useful (10–13). Therefore, we constructed a comprehensive online database of fungal virulence factors for public use to fulfil this important need.

## Database generation and its content

The state-of-the-art text-mining technique is used by the PubMed database and the Internet by searching keywords, such as fungal virulence factors, pathogenic genes and so forth. An in-house tool, programed in Python, is used to fetch article titles and abstracts from the PubMed database, and algorithms used by MedTAKMI (14) are implemented locally for entity extraction. Human intelligence is also involved to screen the output of automatic literature mining methods. Virulence factors are protein products of virulence genes, which are helpful for induction and development of disease (7, 15), but this database focuses on genes and their protein products. Some fungi exist in normal human body flora, such as *Saccharomyces cerevisiae*, and are normally non-pathogenic. However, they could also cause life-threatening infections, which often occur in immunocompromised patients or vulnerable population with weakened immune systems (16, 17). Therefore, virulence factors from this kind of fungi are also included in the database. As a result, 2058 fungal virulence factors are collected, which belong to 85 fungal genera, 228 fungal strains (by NCBI taxonomy ID). These virulence factors come from 593 peer-reviewed journal articles and sequences submitted to GenBank or UniProt databases. Although they are taxonomically different from fungi, oomycetes, as originally being classified among the fungi (18–20), are included this database, and only 79 records come from oomycetes. Of the 2058 proteins, 320 virulence factors are predicted to be secreted by fungi using WoLFPSORT (21) and signalp (22), whereas ∼30 proteins are related to biosynthetic fungal toxin, such as mycotoxin. As a comparison, there are 600 fungal pathogenic genes in the PHI-database (9).

The related information of all virulence factors, such as gene symbol, NCBI database IDs, taxonomy and the protein sequence, is collected. In database of fungal virulence factors (DFVF), the fungus strain taxonomy is recorded with its original reference, as this method may help investigators find the references. In addition, the Pfam (23) domain annotation of each virulence factor and their Gene Ontology annotation are also recorded. The phenotypic information, such as disease information and host, is also collected. In DFVF, all plant diseases and host descriptions are from the US Department of Agriculture database records. Table 1 shows the statistics of hosts and their pathogenic fungi as well as virulence factors in DFVF. For example, there are 1308 virulence factors from 71 pathogens, whose hosts are all vertebrata animals, whereas 539 factors from 65 fungal pathogens have hosts of herbal plants.

**Table 1:** Statistics of virulence factors as their hosts

Host	Genus	Species	Factors
Animal			
All^a^	2	25	451
Vertebrata	36	71	1308
Invertebrata	5	8	45
Plant			
All^a^	23	90	346
Herb	20	65	539
Xyloid	17	67	261

^a^All means that the hosts of the virulence factors have a broad spectrum and cover many different kinds of hosts.

## USER interface

### Search

**Figure 1:**
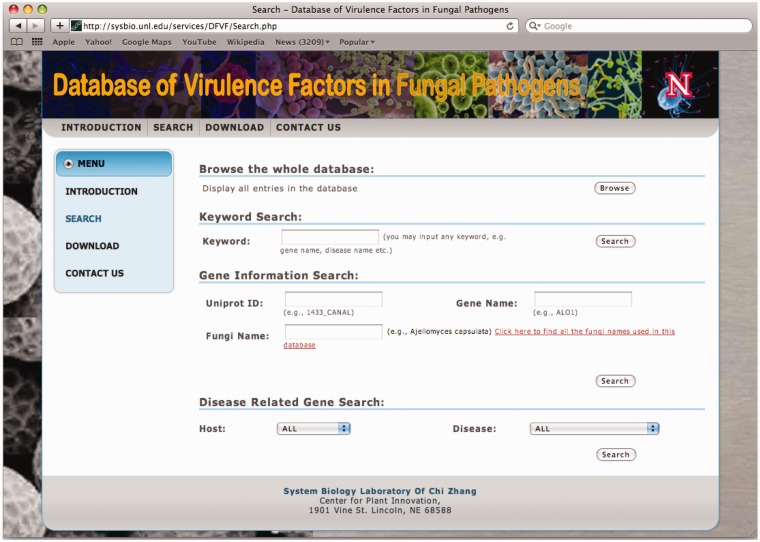
The searching page of the database.

The database system provides interactive access to all of the collected data, and users may connect to the database using a web browser. Figure 1 shows a snapshot of the user interface for users to browse or search the database. The ‘Browse’ button allows users to get a list of all records in one table. Variable search options are provided to conveniently locate the genes of interest. If a user knows the UniProt ID, gene name or a fungus name, the gene information search can be directly applied. If a user wants to get all virulence factors related to a certain disease or host, a disease related gene search can also be conducted. The most convenient searching approach is the keyword search in which the database will return all virulence factors for which the keyword is contained in any piece of the information under the factors.

## Results

**Figure 2:**
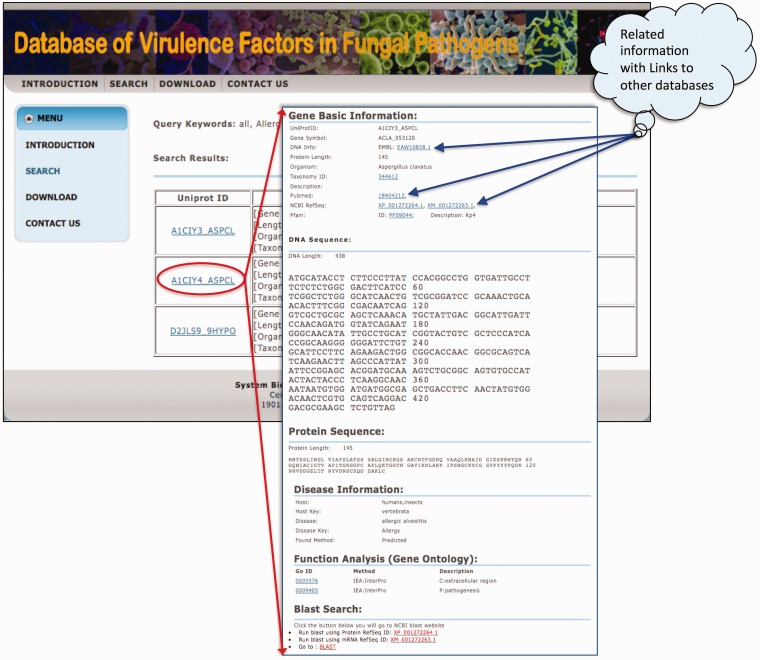
The display page of searching result and information of each gene.

Once a user browses the whole database or searches with a specific option, the database first returns a table of related records, and then displays the UniProt ID, gene information and disease information in three columns, shown in Figure 2. The details of each factor can be displayed by clicking on the link of UniProt ID. The information for each virulence factor includes the basic information, DNA/protein sequences, disease information and ***Gene Ontology*** annotation. The basic information consists of UniProt ID, gene symbol, taxonomy, ID and links to other databases and description. The PubMed links to all related publications are also presented. If the protein of a virulence factor has one or more Pfam domains, the links to those domains are provided. Descriptions on phenotypic data, including diseases and hosts are shown in the section of ‘Disease information***’***.

## Implementation

We adopted the LAMP (Linux, Apache, MySQL, PHP) platform to construct the online database system. The user interface has been designed using the JavaScript application framework. The user interface additionally accepts parameters through a URL for direct searching. This feature facilitates a link to the database from external sites, and it also allows users to bookmark and to cite specific results.

## Accessibility

The database is freely available to all users without restriction at http://sysbio.unl.edu/DFVF. All data are downloadable from the same website. In addition to the link to download the whole database, we provide various links to download data in different categories for convenience. The source codes and other detailed information are available on request.

## Funding

This work is supported by the University of Nebraska—Lincoln start-up funds (to C.Z.) and the Nebraska Soybean Board Funds (to C.Z.). Funding for open access charge: University of Nebraska - Lincoln start-up funds.

*Conflict of interest*. None declared.
